# Beet supplementation mitigates post-exercise inflammation

**DOI:** 10.3389/fnut.2024.1408804

**Published:** 2024-05-30

**Authors:** David C. Nieman, Camila A. Sakaguchi, James C. Williams, Fayaj A. Mulani, Patil Shivprasad Suresh, Ashraf M. Omar, Qibin Zhang

**Affiliations:** ^1^Human Performance Laboratory, Appalachian State University, North Carolina Research Campus, Kannapolis, NC, United States; ^2^UNCG Center for Translational Biomedical Research, University of North Carolina at Greensboro, North Carolina Research Campus, Kannapolis, NC, United States

**Keywords:** beets, exercise, proteomics, oxylipins, inflammation

## Abstract

**Objectives:**

This study investigated the efficacy of a mixed beet-based supplement (BEET) versus placebo (PL) in countering inflammation during recovery from 2.25 h of intensive cycling in 20 male and female cyclists. A multi-omics approach was used that included untargeted proteomics and a targeted oxylipin panel.

**Methods:**

A randomized, placebo-controlled, double-blind, crossover design was used with two 2-week supplementation periods and a 2-week washout period. Supplementation periods were followed by a 2.25 h cycling bout at close to 70%VO_2max_. The BEET supplement provided 212 mg of nitrates per day, 200 mg caffeine from green tea extract, 44 mg vitamin C from Camu Camu berry, B-vitamins from quinoa sprouts (40% Daily Value for thiamin, riboflavin, niacin, and vitamin B6), and 2.5 g of a mushroom blend containing Cordyceps sinensis and Inonotus obliquus. Six blood samples were collected before and after supplementation (overnight fasted state), immediately post-exercise, and at 1.5 h-, 3 h-, and 24 h-post-exercise.

**Results:**

The 2.25 h cycling bout increased plasma levels of 41 of 67 oxylipins detected. BEET supplementation significantly increased plasma nitrate (NO_3_^−^) and nitrite (NO_2_^−^) (sum, NO_3_^−^ + NO_2_^−^) concentrations (interaction effect, *p* < 0.001) and two anti-inflammatory oxylipins [18-hydroxyeicosapentaenoic acid (18-HEPE) and 4-hydroxy-docosahexanoic acid (4-HDoHE)]. The untargeted proteomics analysis identified 616 proteins (458 across all times points), and 2-way ANOVA revealed a cluster of 45 proteins that were decreased and a cluster of 21 that were increased in the BEET versus PL trials. Functional enrichment supported significant BEET-related reductions in inflammation-related proteins including several proteins related to complement activation, the acute phase response, and immune cell adhesion, migration, and differentiation.

**Discussion:**

Intake of a BEET-based supplement during a 2-week period was linked to higher plasma levels of NO_3_^−^ + NO_2_^−^, elevated post-exercise levels of two anti-inflammatory oxylipins, and a significant decrease in a cluster of proteins involved in complement activation and inflammation. These data support that 2-weeks intake of nitrate from a mixed beet-based supplement moderated protein biomarkers of exercise-induced inflammation in athletes.

## Introduction

Nitrate is found in some root vegetables such as beets and in green leafy vegetables such as spinach and lettuce. The typical Western diet provides about 110 mg/day nitrate (1, 2). After ingestion, nitrate is metabolized to nitric oxide and other bioactive nitrogen oxides through a complex pathway. Nitrate is absorbed in the small intestine and peak plasma levels can be measured 30–60 min post-intake with an effective half-life of about six hours (3). Nitrate is cleared by the kidneys, but about 25% of the nitrate is taken up and secreted by the salivary glands and is subsequently reduced to nitrite by commensal bacteria in the mouth (4). The nitrite is then swallowed, absorbed through the intestinal tract, and further reduced to nitric oxide and other nitrite intermediates by enzymatic and nonenzymatic mechanisms in the blood and tissues. The use of mouthwashes, antacids, and chewing gum can interfere with the microbiome in the mouth and the conversion of nitrate to nitrite.

Dietary nitrate has been linked to several health-related benefits including blood pressure reduction, improved vascular function, and modulation of inflammatory processes and immune cell function (3). Nitric oxide acts as a signaling molecule during exercise and has physiological effects including vasodilation to increase blood flow regulation of muscle contraction and glucose uptake regulation of cellular respiration. The primary performance benefit of nitrate supplementation appears to be a reduction in the energy cost of exercise and a corresponding modest improvement in endurance capacity (5–7). Studies reporting performance benefits used acute nitrate doses of 5–6 mmol (or about 300 mg), or chronic doses at about half that amount (8). Chronic nitrate supplementation has not been consistently linked to improvements in aerobic capacity or performance (9). Concerns have been raised about high nitrate intake by athletes and the formation of carcinogenic N-nitroso compounds, a process that may be inhibited by vitamin C (10–13). An acceptable daily intake (ADI) level of 3.7 mg nitrates per kg body weight has been recommended by the Joint FAO/WHO Expert Committee on Food Additives (JECFA) (14). This recommendation includes all nitrate sources including plant foods, drinking water, and cured and processed meats, although a different approach is recommended for nitrate from plant food sources (15). Co-supplementation of nitrate with caffeine, vitamin C, and other food components may enhance potential beneficial effects from moderate intake of nitrate and minimize risks from N-nitroso compounds (16).

Nitric oxide is considered a potent anti-inflammatory mediator and inhibitor of leukocyte recruitment, a key feature of inflammatory responses (3). However, most of the evidence comes from rodent-based studies. Human studies investigating the effects of nitrate or nitrate-rich beetroot juice on exercise-induced inflammation and muscle damage have produced mixed results (8, 17–19). Part of the problem is that only a few basic outcome measures related to inflammation (e.g., C-reactive protein, IL-6, IL-8) have been utilized in these studies. This study used a multi-omics approach centered on targeted oxylipins and untargeted proteomics analysis to investigate the influence of 2-weeks dietary nitrate ingestion from a beet-based mixed supplement on exercise-induced inflammation. Oxylipins are upstream regulators of inflammation, increase strongly after prolonged and vigorous exercise, and exert both pro- and anti-inflammatory effects depending on the fatty acid substrate and enzyme system (20–24). Untargeted proteomics studies indicate that long endurance cycling and running cause perturbations in numerous immune system and inflammatory proteins (25–27). Previous studies by our research group indicate that exercise-induced changes in inflammatory oxylipins and proteins can be moderated through nutrition-based interventions (21–24, 28, 29). We hypothesized that a multi-omics approach would reveal that 2-weeks ingestion of a beet-based supplement would mitigate exercise-induced inflammation.

## Methods

### Study participants

Male and female cyclists were invited to take part in this study if they met the inclusion criteria including 18 to 60 years of age, capable of cycling 2.25 h in a laboratory setting at 70% maximal oxygen consumption rate (VO_2max_), and a willingness to avoid supplements and medications such as non-steroidal anti-inflammatory drugs (NSAIDs) with a potential to influence inflammation. Participants also agreed to limit intake of nitrate-rich vegetables during the study to less than 1 cup per day. These vegetables included spinach, lettuce, beets, beetroot juice, celery, and cabbage. Participants also agreed to avoid the use of mouthwashes, antacids, and chewing gum during the entire 6-week study and the 2-week period prior to the study. During the 3-day period prior to the 2.25 h cycling session, subjects agreed to taper exercise training and ingest a moderate-carbohydrate diet using a food list restricting high fat foods and visible fats.

**Figure 1:**
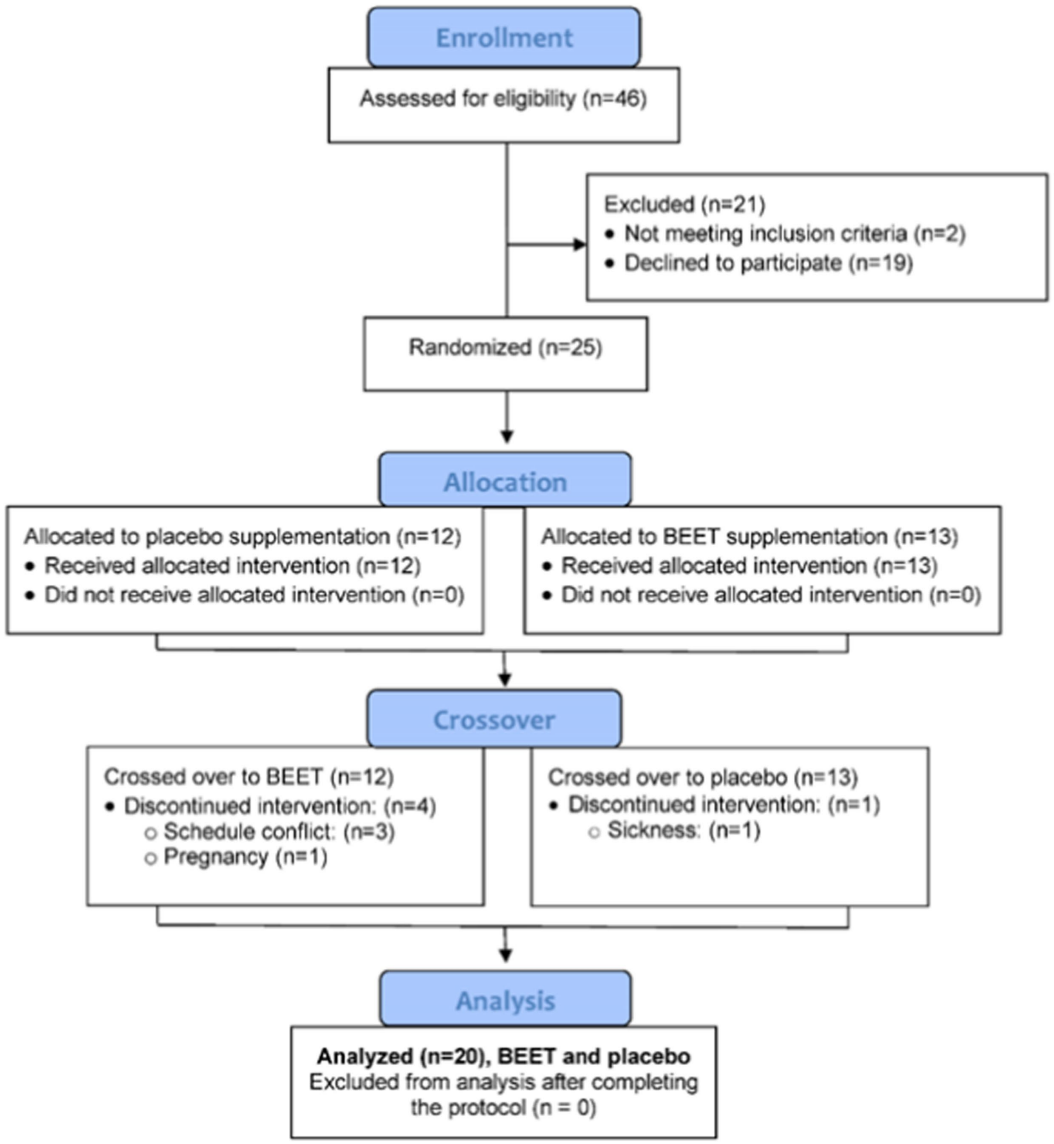
Study participant flow diagram.

A total of 46 participants were assessed for eligibility and 25 were entered into the study, with 20 completing all aspects of the protocol (Figure 1). The study participant number provided more than 80% power to detect a difference in pro-inflammatory oxylipins with an effect size 1.06 at alpha 0.05 using two-sample *t*-tests (23). Participants voluntarily signed the informed consent, and procedures were approved by the university’s Institutional Review Board. Trial Registration: ClinicalTrials.gov, U.S. National Institutes of Health, identifier: NCT05907135.

### Study design

This study employed a randomized, placebo controlled, double-blind, crossover design with two 2-week supplementation periods and a 2-week washout period. The study included seven lab visits at the Appalachian State University Human Performance Laboratory (HPL) at the North Carolina Research Campus, Kannapolis, NC.

During the first two lab visits prior to the 2-week supplementation period, study participants were given a complete orientation to the study protocol, signed the consent form, provided an overnight fasted blood sample, reported demographics and training histories using questionnaires, and recorded responses to the delayed onset of muscle soreness (DOMS) 1–10 scale questionnaire (30). Height and body weight were assessed, with body composition measured using the BodPod system (Cosmed, Rome, Italy). Study participants were tested for maximal aerobic capacity (VO_2max_) during a graded, cycling test with the Lode cycle ergometer (Lode B.V., Groningen, Netherlands) and the Cosmed CPET metabolic cart (Cosmed, Rome, Italy).

The beet-based (BEET) and placebo (PL) supplements (randomized order using double-blind procedures) for the first and second 2-week supplementation periods were supplied in coded packets. To facilitate compliance to the supplementation protocol, study participants were contacted via email on a regular basis and also returned the coded packets at the end of the supplementation period. Supplements ingested daily (two doses, early morning and mid-day, each mixed in 250 mL cold water) for 2-weeks prior to participation in the first 2.25 h cycling session. After a 2-week washout period, participants repeated all procedures using the counterbalanced supplement. The BEET and PL supplements were supplied by the sponsor (Standard Process, Palmyra, Wisconsin, USA). Each BEET packet (14 grams) included powder from beets and fermented beets to provide nitrates (106 mg of nitrates per serving), green tea extract with caffeine (100 mg), Camu Camu to provide vitamin C (22 mg), quinoa sprouts to provide B-vitamins (20% of the Daily Value for thiamin, riboflavin, niacin, and vitamin B6), and a mushroom blend of three common mushrooms (1.5 g of caterpillar fungus, lion’s mane, chaga). The PL supplement included excipients with none of the active ingredients. PL ingredients included a carbohydrate blend (monosaccharides, disaccharides, maltodextrin, corn starch), monk fruit extract, dietary fibers including cellulose and rice bran, rice silica, malic acid, beet flavor, cranberry flavor, and colorants. Participants reported no adverse events from ingesting the supplements over the 2-week periods and were 100% compliant with the supplementation regimen. After the study was completed, study participants responded to a questionnaire regarding “what supplement do you think you were taking during each of the 2-week supplementation periods?” A total of 22.5 and 60% correctly identified the use of PL and BEET supplements, respectively (Chi-square = 12.95, *p* = 0.0015). Study participants reported a red color in their urine and stool when using the BEET supplement.

After the 2-week supplementation period, study participants reported to the Human Performance Lab in an overnight fasted state, provided a blood sample, ingested one 14-gram packet of the BEET or PL supplement with 250 mL water, and then cycled for 2.25 h at high intensity (70% VO_2max_) while ingesting water alone (3 mL/kg every 15 min). Participants cycled on their own bicycles fitted to Saris H3 direct drive smart trainers (Madison, WI, USA) with monitoring by the Zwift online training platform (Long Beach, CA, USA). Heart rate, cycling speed, cadence, distance, power, breathing rate, ventilation, and oxygen intake were measured after 15 min and then every 30 min during the cycling session. To ensure performance consistency between trials and to focus on the effect of the BEET supplement on exercise-induced inflammation, performance data from the first trial were used to ensure a similar power and metabolic output during the second trial. Blood samples were collected at 0 h, 1.5 h, 3 h, and 24 h post-exercise. After each blood collection, participants provided a DOMS rating. Immediately after the 1.5 h post-exercise blood sample, all subjects consumed 7 kilocalories per kilogram of body weight of a fortified nutrient beverage (Boost, Nestlé S.A., Vevey, Switzerland). Blood samples were aliquoted and stored at-80°C prior to analysis for the outcome measures.

### Sample analysis

Serum creatine kinase, myoglobin, and coritsol (from serum separator tubes), and complete blood counts (CBCs) with a white blood cell differential count (EDTA tubes) were analyzed using Labcorp services (Burlington, NC). Plasma aliquots were prepared from EDTA blood collection tubes and stored in a-80°C freezer until analysis for nitrate, nitrite, proteomics, and oxylipins after the study was completed.

#### Plasma nitrate and nitrite

The nitrate/nitrite fluorometric assay kit (item # 780051) from the Cayman Chemical Company (Ann Arbor, MI) was used to measure plasma concentrations of nitrate (NO_3_^−^) and nitrite (NO_2_^−^). This assay was performed in accordance with the instructions provided by the manufacturer with the plates read using the SpectraMax iD3 Multi-Mode Microplate Reader (Molecular Devices, LLC San Jose, CA, USA). Data were processed using BioTek Gen5 2.0 software (BioTek, Agilent, Santa Clara, CA, USA). Samples were analyzed in duplicate (mean coefficient of variation or CV of 9.8%). The sum of both NO_3_^−^ and nitrite NO_2_^−^ was used as an index of total nitric oxide production as recommended by Cayman (lower limit of detection 0.70 μM, intra-assay CV 2.7%). The process includes 2 steps: conversion of nitrate to nitrite using a nitrate reductase, followed by addition of an acidic solution (DAN) which isolates nitrate and yields the product 1(H)-naphthotriazole. Sodium hydroxide (NaOH) is then added to enhance fluorescence of the product. Sample concentrations were plotted along a standard curve, which was made using fresh standards provided in the assay kits.

#### Plasma oxylipins

Plasma arachidonic acid (ARA), eicosapentaenoic acid (EPA), docosahexaenoic acid (DHA), and oxylipins were analyzed using a liquid chromatography-multiple reaction monitoring mass spectrometry (LC-MRM-MS) method as fully described elsewhere (31). Resultant data files were processed with Skyline, and the auto-integrated peaks were inspected manually. Concentrations of each oxylipin were determined from calibration curves of each analyte, which were constructed by normalizing to the selected deuterated internal standards followed by linear regression with 1/x weighting (Supplement Data Sheet 1). The coefficient of variation for the quality control standards was <15% as reported in the method development paper (31). A total of 41 of 67 oxylipins detected increased significantly post-exercise, and these were grouped for statistical analysis. Seven oxylipins generated from arachidonic acid and cytochrome P-450 (ARA-CYP) were grouped and these included 5,6-, 8,9-, 11,12-, and 14,15-dihydroxy-eicosatetraenoic acid (diHETrEs), 16-, 17-hydroxy-eicosatetraenoic acids (HETEs), and the 20-HETE metabolite 20-carboxy-arachidonic acid (20-coohAA). Four abundant oxylipins generated from linoleic acid (LA) with CYP and lipoxygenease (LOX) were also grouped, including 9,10- dihydroxy-9Z-octadecenoic acid (DiHOME), 12,13-DiHOME, 9- hydroxy-octadecadienoic acid (HODE), and 13-HODE (LA-DiHOMES + HODES).

#### Plasma proteome and statistical procedures

Untargeted proteomics were conducted using methods previously described (22, 32). Briefly, after sample preparation, 200 ng peptides from the plasma samples were loaded onto disposable EvoTip trap-columns (EV-2003, EvoSep, Denmark) and separated on an EvoSep One ™ LC system (EV-1000, EvoSep, Denmark) using a 21 min gradient with 1 μL/min flow rate. Effluents were analyzed on a high resolution Orbitrap Exploris 240 (Thermo) mass spectrometer using the data independent acquisition (DIA) method. The plasma protein library was generated using the gas-phase fractionation DIA method from peptide samples with and without depletion of the top 14 high-abundance plasma proteins (14,120 precursors, 960 proteins). The obtained LC–MS/MS dataset was searched for protein identification and quantitation using DIA-NN (33). Data were normalized by referencing to the protein levels of the first time point from the same individual subject to effectively correct for inter-individual variations (34) (Supplementary Data Sheet 2). The normalized values were statistically analyzed using the ANOVA test with two trials and six timepoints. To consider the protein as significantly changing between or within effects, the *p*-value was set to less than 0.05. Maximum likelihood-based hierarchical clustering analysis was used to cluster proteins with similar level patterns, and the results were visualized as a heatmap with the averaged value of each time-point after normalization by z-score. The list of significantly changed proteins in the enriched clusters were functionally enriched using STRING (Ver.11.5).^1^ The top enriched biological processes from STRING analysis were selected to represent the functions of the proteins.

### Additional statistical procedures

The data are expressed as mean ± SE and were analyzed using the generalized linear model (GLM), repeated measures ANOVA module in SPSS (IBM SPSS Statistics, Version 28.0, IBM Corp, Armonk, NY, USA). The statistical model utilized the within-subjects approach: 2 (trials) x 6 (time points) repeated measures ANOVA and provided time (i.e., the collective effect of the cycling exercise bout) and interaction effects (i.e., whether the data pattern over time differed between trials). If the interaction effect was significant (*p* ≤ 0.05), then post-hoc analyses were conducted using paired *t*-tests comparing time point contrasts between trials. An alpha level of *p* ≤ 0.01 was used after Bonferroni correction for 5 multiple tests. The positive false discovery rate (FDR or “q-value”) was calculated for multiple testing correction of the plasma oxylipin and plasma proteomics data. Cohen’s d effect size was calculated for the oxylipin data as the mean difference between the trials at the immediate post-exercise time point divided by the pooled standard deviation.

## Results

Table 1 summarizes characteristics for the *n* = 20 study participants (*n* = 14 males, *n* = 6 females) who completed the study protocol. Age, percent body fat, aerobic capacity (VO2max), and training volume were similar between the male and female cyclists. The pattern of change over time did not differ between the male and female cyclists for a key outcome measurement (total plasma oxylipins, supplement x time x sex interaction effect, *p*-value =0.835). Thus, outcome measures for this randomized, crossover study are presented for all participants combined.

**Table 1:** Subject characteristics (*n* = 20) for male (*n* = 14) and female (*n* = 6) cyclists.

	Sex	Mean	SE
Age (years)	M = male	46.5	2.6
	F = female	51.3	4.3
Body mass (kg)	M	80.5*	3.5
	F	60.2	2.6
Height (cm)	M	179*	1.6
	F	167	1.6
Body mass index (BMI) (kg/m^2^)	M	25.0*	0.9
	F	21.7	1.0
Body fat (%)	M	19.5	1.8
	F	25.0	3.7
Maximum oxygen consumption (VO_2max_) (mL^.^kg.^−1^ min^−1^)	M	41.2	1.6
	F	40.9	2.9
Maximum heart rate (beats/min)	M	170	4.0
	F	160	3.8
Maximum watts	M	282*	12.4
	F	217	20.1
Cycling training distance (km/wk)	M	124	18.2
	F	139	31.1

*p ≤ 0.05.

Performance data for each trial are summarized in Table 2. As designed, the two trials were similar in all cycling performance measures including total distance cycled, and percent of maximal heart rates, watts, and oxygen consumption rates.

**Table 2:** Average 2.25 h cycling performance outcomes for *n* = 20 cyclists (mean ± SE).

Performance measurement		Mean ± SE
Cycling power (watts)	PL	161 ± 6.9 (61.9 ± 1.4% max)
	BEET	164 ± 6.8 (63.0 ± 1.3% max)
Heart rate (beats/min)	PL	131 ± 2.6 (78.9 ± 1.7% max)
	BEET	132 ± 2.8 (80.2 ± 1.8% max)
Oxygen consumption rate (VO_2_) (ml^.^kg^.-1^ min^−1^)	PL	30.1 ± 1.1 (73.6 ± 2.1% max)
	BEET	31.7 ± 1.1 (77.5 ± 2.2% max)
Respiratory exchange ratio (RER)	PL	0.834 ± 0.001
	BEET	0.817 ± 0.007
Distance cycled (km)	PL	64.9 ± 1.6
	BEET	65.7 ± 1.7
Elevation gain (m)	PL	314 ± 33.0
	BEET	282 ± 24.2
Speed (km/h)	PL	27.7 ± 1.1
	BEET	29.1 ± 0.6
Cycling cadence (pedal revolutions/min)	PL	71.4 ± 2.9
	BEET	74.0 ± 1.8
Weight change (kg)	PL	1.13 ± 0.11
	BEET	1.08 ± 0.13

No significant placebo (PL) vs. BEET trial differences in performance measurements were found as designed.

Data for delayed onset of muscle soreness (DOMS), muscle damage biomarkers, the neutrophil/lymphocyte ratio, and serum cortisol are summarized in Table 3. Time effects for each of these variables were significant but the patterns of increase did not differ significantly between the BEET and PL trials (interaction effects, *p*-values >0.05).

**Table 3:** Trial comparisons for *n* = 20 participants across all time points for muscle soreness* and damage markers (serum creatine kinase and myoglobin), the neutrophil/lymphocyte blood count ratio, and serum cortisol.

Variable	Trial	Pre- Study	2-Wks Suppl.	0 h Post-Ex	1.5 h Post-Ex	3 h Post-Ex	24 h Post-Ex	*p*- value
DOMS (1–10 scale)	PL	1.7 ± 0.3	1.7 ± 0.2	4.1 ± 0.4	3.6 ± 0.4	2.7 ± 0.3	1.9 ± 0.2	<0.001; 0.069
	BEET	2.1 ± 0.4	1.6 ± 0.2	4.2 ± 0.4	3.3 ± 0.4	2.5 ± 0.3	2.1 ± 0.2	
Creatine kinase (U/L)	PL	143 ± 14.4	141 ± 17.1	177 ± 24.3	166 ± 21.4	172 ± 22.9	158 ± 22.3	0.008; 0.974
	BEET	174 ± 21.4	174 ± 23.7	279 ± 83.1	266 ± 81.9	266 ± 77.8	229 ± 50.5	
Myoglobin (ng/ml)	PL	35.5 ± 2.9	32.4 ± 1.9	47.4 ± 3.9	58.1 ± 10.0	52.0 ± 9.2	36.7 ± 3.1	0.002; 0.595
	BEET	40.1 ± 3.5	34.1 ± 2.1	57.0 ± 6.6	67.0 ± 6.8	63.7 ± 9.3	38.0 ± 3.6	
Neutrophil/lymphocyte	PL	1.6 ± 0.1	1.5 ± 0.1	4.0 ± 0.4	5.2 ± 0.5	4.9 ± 0.5	1.7 ± 0.2	<0.001; 0.163
	BEET	1.6 ± 0.2	1.6 ± 0.2	4.2 ± 0.5	6.1 ± 0.7	5.5 ± 0.6	1.8 ± 0.2	
Cortisol (μg/dl)	PL	14.0 ± 0.8	15.4 ± 0.8	16.9 ± 1.2	11.8 ± 1.0	9.7 ± 0.6	14.3 ± 0.9	<0.001; 0.682
	BEET	13.8 ± 0.8	14.8 ± 0.8	18.3 ± 1.4	13.3 ± 1.1	11.0 ± 0.8	14.7 ± 0.7	

*P*-values represent time (first value) and trial x time interaction effects. ^*^Ex, exercise; Suppl, supplement; PL, placebo; DOMS, delayed onset of muscle soreness.

**Figure 2:**
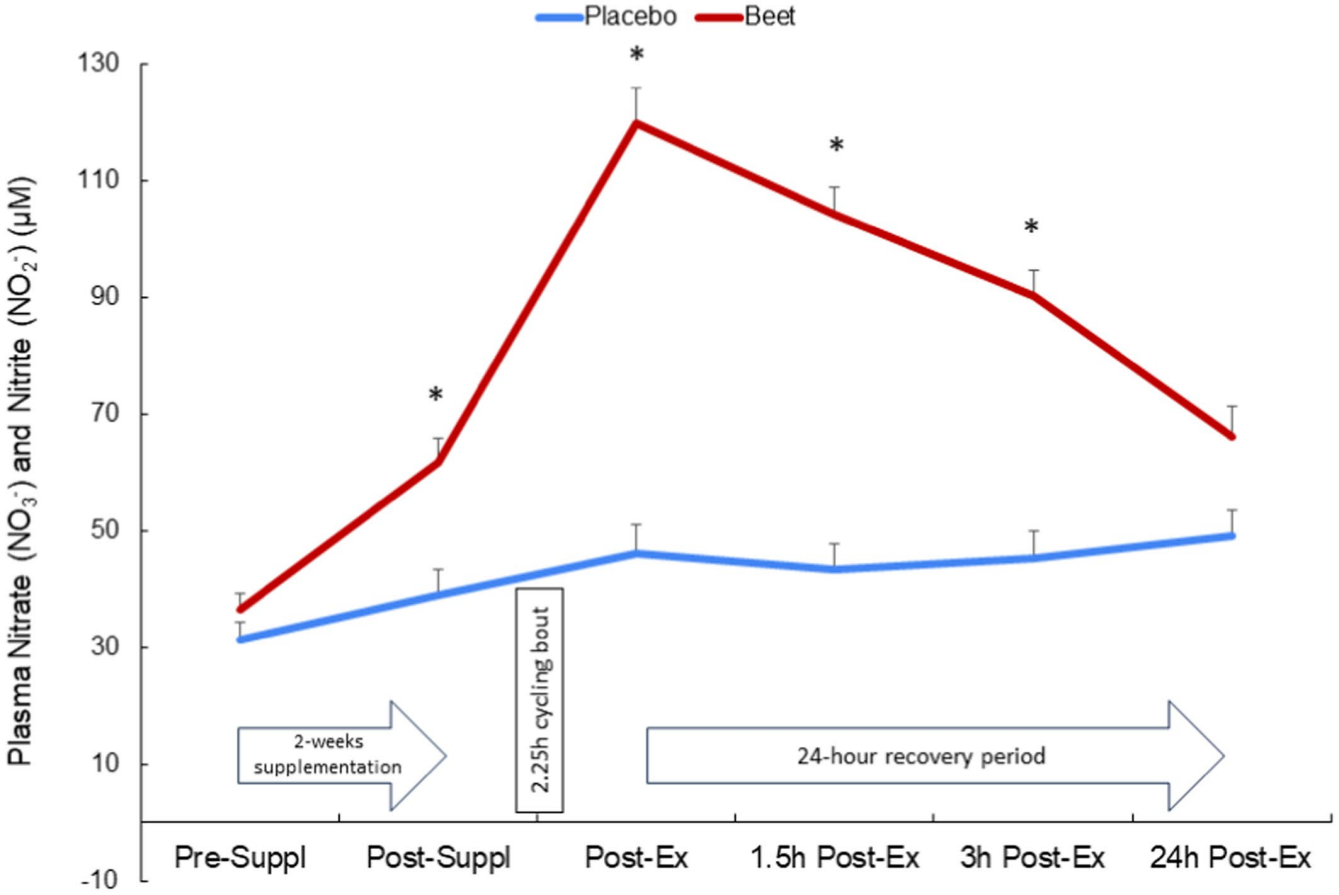
Change in the sum of plasma nitrate and nitrite after 2-weeks BEET and placebo supplementation and during 24 h recovery from 2.25 h cycling in *n* = 20 cyclists. Time and interaction effects, both *p* < 0.001.

BEET compared to placebo intake was associated with a significant increase in the sum of plasma nitrate and nitrite (NA + NI) (time and interaction effects, both *p* < 0.001) (Figure 2). The increase in NA + NI was significantly different between the BEET and PL trials after 2-weeks supplementation and throughout at least 3 h of recovery from the cycling bout. Trial differences for NA + NI at 24 h post-exercise tended to be different (*p* = 0.089).

**Figure 3:**
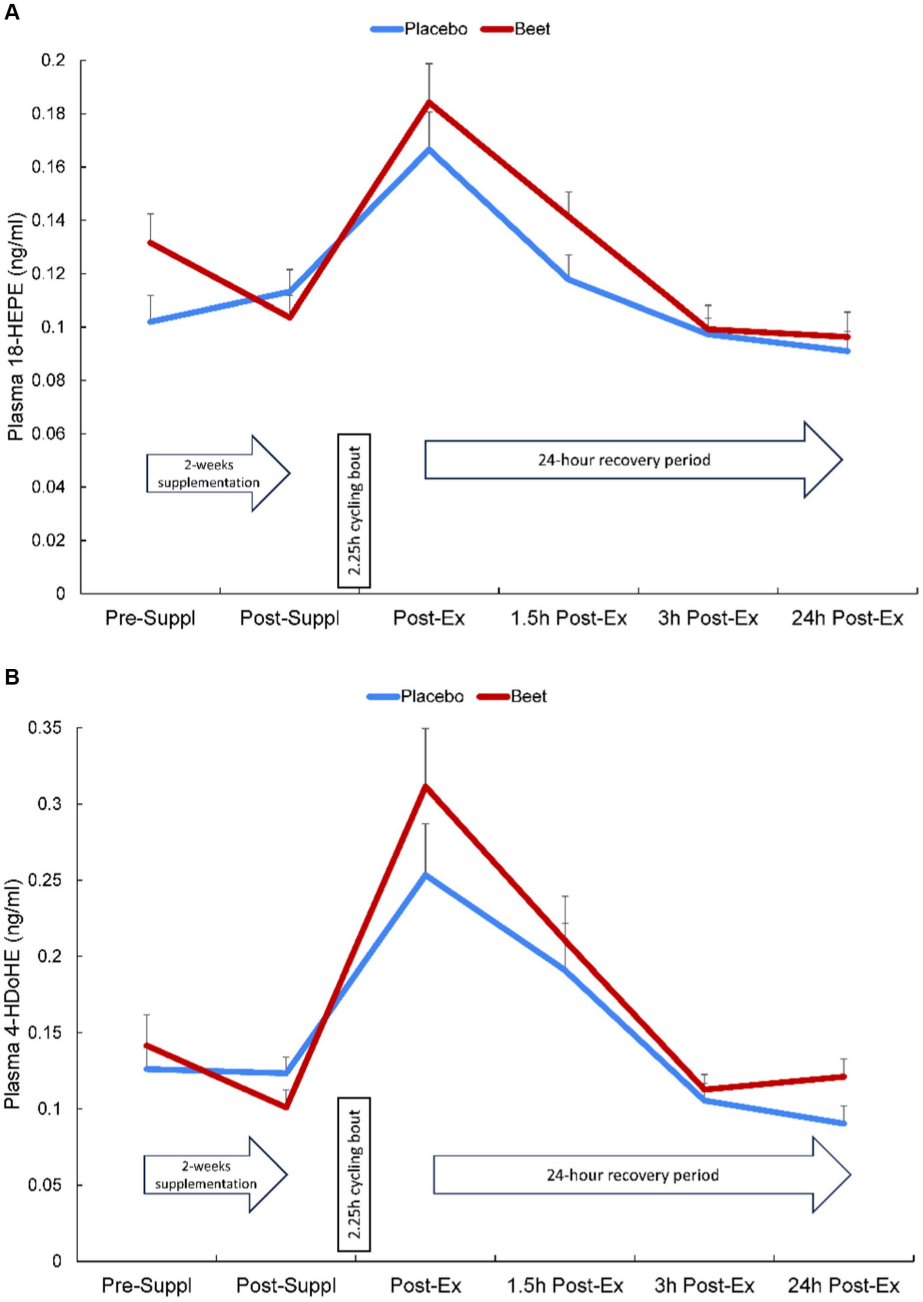
Change in **(A)** plasma HEPE-18 and **(B)** 4-HDoHE in BEET and placebo trials during 24 h recovery from 2.25 h cycling in *n* = 20 cyclists. Interaction effect, *p* = 0.016 and 0.010, respectively.

Plasma arachidonic acid (ARA), docosahexaenoic acid (DHA), and eicosapentaenoic acid (EPA) increased significantly post-exercise (time effects, *p* < 0.001) with no significant interaction effects (all *p* > 0.50) (Supplementary Data Sheet 1). A total of 67 oxylipins were detected in study samples. Of these, 41 oxylipins increased significantly post-exercise (Supplementary Data Sheet 1). These 41 oxylipins were summed for a composite variable and analysis showed significant post-exercise increases without trial differences between BEET and PL (interaction effect, *p* = 0.166) (Cohen’s *d* = 0.219, immediately post-exercise) (Table 4). Three other composite variables were calculated including seven oxylipins generated from arachidonic acid and cytochrome P-450 (ARA-CYP), two abundant oxylipins generated from linoleic acid and CYP (9,10-DiHOME, 12,13-DiHOME), and two abundant oxylipins generated from lipoxygenase (LOX) (9-HODE, 13-HODE) (Table 4). Significant time effects were shown for each of these composite oxylipin variables but without significant interaction effects (Table 4) (Cohen’s *d* = 0.102, 0.221, 0.283, respectively, immediately post-exercise). Two anti-inflammatory oxylipins were significantly higher in the BEET versus PL trials (Figures 3A,B). These included the EPA-derived oxylipin 18-hydroxyeicosapentaenoic acid (18-HEPE) (time effect, *p* < 0.001, interaction effect, *p* = 0.016) and the DHA-derived oxylipin 4-hydroxydocosahexaenoic acid (4-HDoHE) (time effect *p* < 0.001, interaction effect, *p* = 0.010).

**Table 4:** Trial comparisons across all time points for total plasma oxylipins and oxylipin subgroups.

Variable (ng/mL)	Trial	Pre- Suppl.	2-Wks Suppl.	0 h Post-Ex	1.5 h Post-Ex	3 h Post-Ex	24 h Post-Ex	*p*- value
Oxylipins, total (*n* = 41 oxylipins)	PL	21.3 ± 1.7	24.1 ± 2.9	67.3 ± 6.2	35.0 ± 3.3	32.5 ± 8.3	19.3 ± 1.2	<0.001; 0.166
	BEET	21.2 ± 1.7	20.5 ± 1.4	76.4 ± 11.6	37.7 ± 4.4	31.9 ± 8.7	20.3 ± 1.3	
DiHOMES (9,10 + 12,13)	PL	3.2 ± 0.5	3.8 ± 0.7	10.4 ± 1.0	3.9 ± 0.6	5.9 ± 2.7	2.5 ± 0.2	<0.001; 0.255
	BEET	3.1 ± 0.5	2.6 ± 0.3	11.6 ± 1.4	3.6 ± 0.6	6.6 ± 3.4	2.7 ± 0.3	
HODES (9 + 13)	PL	5.7 ± 0.9	7.6 ± 1.5	25.7 ± 2.6	8.9 ± 1.2	9.3 ± 4.9	4.7 ± 0.4	0.002; 0.094
	BEET	6.0 ± 1.0	5.2 ± 0.4	31.8 ± 6.3	9.1 ± 1.9	7.9 ± 4.4	5.0 ± 0.5	
ARA-CYP* (*n* = 7 oxylipins)	PL	4.5 ± 0.3	4.6 ± 0.3	8.4 ± 0.8	10.6 ± 1.0	8.8 ± 0.9	4.9 ± 0.3	<0.001; 0.163
	BEET	4.5 ± 0.4	4.7 ± 0.4	9.3 ± 1.0	13.6 ± 1.9	10.3 ± 1.4	4.9 ± 0.4	

*p*-values represent time (first value) and trial x time interaction effects. ^*^ARA-CYP = 7 oxylipins generated from arachidonic acid and cytochrome P-450 (ARA-CYP) were grouped and these included 5,6-, 8,9-, 11,12-, and 14,15-dihydroxy-eicosatetraenoic acid (diHETrEs), 16-, 17-hydroxy-eicosatetraenoic acids (HETEs), and the 20-HETE metabolite 20-carboxy-arachidonic acid (20-coohAA).

**Figure 4:**
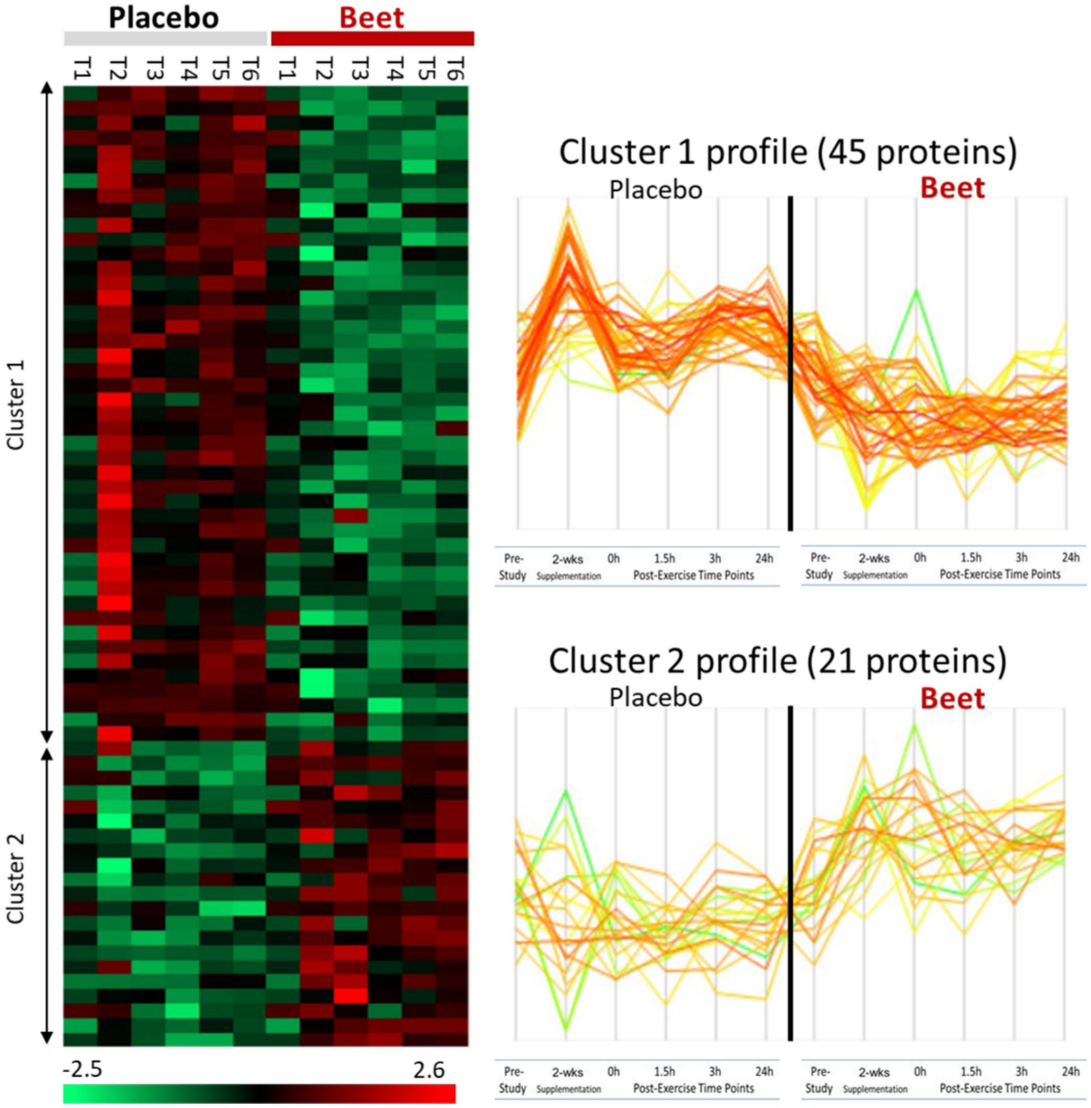
Heatmap and line graphs of clustered proteins in the BEET and placebo trials. T1, pre-study; T2, 2-weeks supplementation, pre-exercise; T3, immediately post-exercise (2.25 h cycling bout); T4, 1.5 h post-exercise; T5, 3 h post-exercise; T6, 24 h post-exercise.

**Figure 5:**
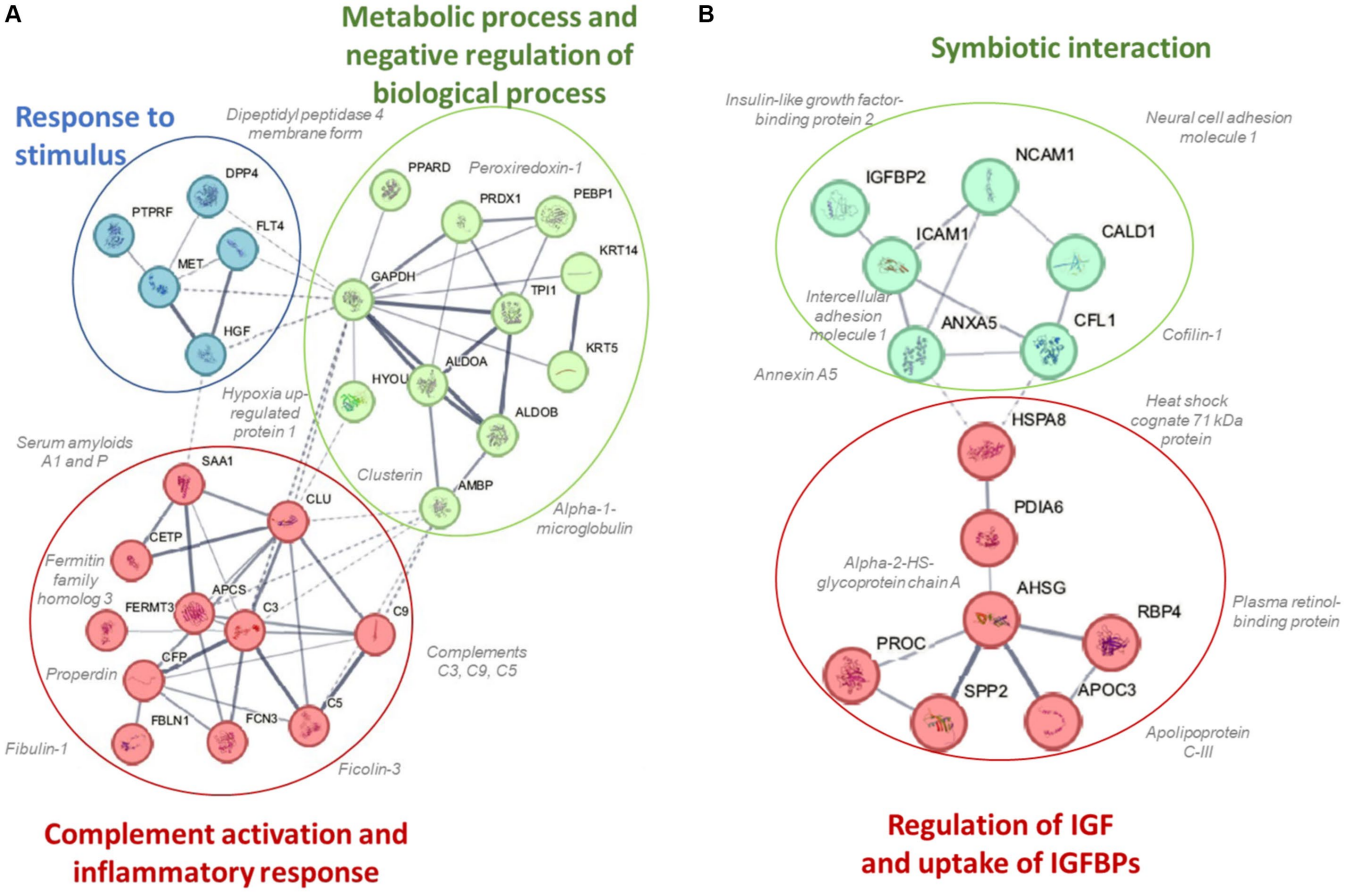
Protein–protein interaction networks and functional enrichments of **(A)** cluster 1 (45 proteins) that were lower and **(B)** cluster 2 (21 proteins) that were higher in the BEET compared to placebo trials.

A total of 616 plasma proteins were identified with no missing data for 458 proteins across all time points (Supplementary Data Sheet 2). Two-way ANOVA analysis of the normalized data showed that 45 proteins were lower and 21 proteins were higher in the BEET versus PL trials (all *p* < 0.05). The heatmap and line graphs of the clustered proteins are shown in Figure 4, and the identity of the clustered proteins is summarized in Table 5. Functional enrichment and protein–protein interaction networks are depicted in Figure 5 and supported a linkage of BEET supplementation with reduced complement activation and inflammatory responses, metabolic process and negative regulation of biological processes, and responses to stimulus, and an increased regulation of insulin-like growth factors (IGF) and uptake of IGF binding proteins, and symbiotic interactions.

**Table 5:** List of 45 proteins that were lower and 21 proteins that were higher with BEET compared to placebo supplementation.

Protein ID	Gene	Protein description
Lower in BEET trial		
Q9BXT5; Q9Y4L1	HYOU1	Hypoxia up-regulated protein 1
Q9ULV4	CORO1C	Coronin-1C
Q96KN2	CNDP1	Beta-Ala-His dipeptidase
Q86UX7	FERMT3	Fermitin family homolog 3
Q6UXB8	PI16	Peptidase inhibitor 16
Q06830	PRDX1	Peroxiredoxin-1
Q03181	PPARD	Peroxisome proliferator-activated receptor delta
P80748	IGLV3-21	Immunoglobulin lambda variable 3–21
P78509	RELN	Reelin
P52566	ARHGDIB	Rho GDP-dissociation inhibitor 2
P40197	GP5	Platelet glycoprotein V
P35916	FLT4	Vascular endothelial growth factor receptor 3
P30086	PEBP1	Phosphatidylethanolamine-binding protein 1
P27918	CFP	Properdin
P27487	DPP4	Dipeptidyl peptidase 4
P23142	FBLN1	Fibulin-1
P19021	PAM	Peptidyl-glycine alpha-amidating monooxygenase
P15531; P22392; O60361	NME1;NME2	Nucleoside diphosphate kinase A
P13727	PRG2	Bone marrow proteoglycan
P13647	KRT5	Keratin, type II cytoskeletal 5
P11597	CETP	Cholesteryl ester transfer protein
P10909	CLU	Clusterin
P10586	PTPRF	Receptor-type tyrosine-protein phosphatase F
P0DJI8; P0DJI9	SAA1	Serum amyloid A-1 protein
P08581	MET	Hepatocyte growth factor receptor
P06312	IGKV4-1	Immunoglobulin kappa variable 4–1
P05062	ALDOB	Fructose-bisphosphate aldolase B
P04406	GAPDH	Glyceraldehyde-3-phosphate dehydrogenase
P04075	ALDOA	Fructose-bisphosphate aldolase A
P02760	AMBP	Alpha-1-microglobulin
P02748	C9	Complement component C9
P02743	APCS	Serum amyloid P-component
P01857; P01859; P0DOX5	IGHG2	Immunoglobulin heavy constant gamma 2
P01857; P01860; P01861; P0DOX5	IGHG1	Immunoglobulin heavy constant gamma 1
P01834; P0DOX7	IGKC	Immunoglobulin kappa constant
P01766	IGHV3-13	Immunoglobulin heavy variable 3–13
P01742	IGHV1-69	Immunoglobulin heavy variable 1–69
P01714	IGLV3-19	Immunoglobulin lambda variable 3–19
O75636	FCN3	Ficolin-3
P0CG04; P0DOY2; P0DOY3; A0M8Q6; P0CF74; B9A064; P0DOX8	IGLL5	Immunoglobulin lambda-like polypeptide 5
P01599; A0A0C4DH72	IGKV1-6	Immunoglobulin kappa variable 1–6
P01814; A0A0C4DH43	IGHV2-70D	Immunoglobulin heavy variable 2-70D
A0A0B4J1X8	IGHV3-43	Immunoglobulin heavy variable 3–43
P01615; A0A075B6P5	IGKV2-28; IGKV2D-28	Immunoglobulin kappa variable 2–28
A0A0B4J1V2	IGHV2-26	Immunoglobulin heavy variable 2–26
Higher in BEET trial		
Q8NBJ4	GOLM1	Golgi membrane protein 1
Q7Z7M0	MEGF8	Multiple epidermal growth factor-like domains protein 8
Q7Z7G0	ABI3BP	Target of Nesh-SH3
Q15084	PDIA6	Protein disulfide-isomerase A6
Q13103	SPP2	Secreted phosphoprotein 24
Q05682	CALD1	Caldesmon
P54289	CACNA2D1	Voltage-dependent calcium channel subunit alpha-2/delta-1
P23528; Q9Y281	CFL1	Cofilin-1
P18065	IGFBP2	Insulin-like growth factor-binding protein 2
P16930	FAH	Fumarylacetoacetase
P13645	KRT10	Keratin, type I cytoskeletal 10
P13591	NCAM1	Neural cell adhesion molecule 1
P0DMV8; P0DMV9; P11142; P17066; P34931; P54652; P48741	HSPA8	Heat shock cognate 71 kDa protein
P08758	ANXA5	Annexin A5
P05362	ICAM1	Intercellular adhesion molecule 1
P04070	PROC	Vitamin K-dependent protein C
P02765	AHSG	Alpha-2-HS-glycoprotein
P02753	RBP4	Retinol-binding protein 4
P02656	APOC3	Apolipoprotein C-III
P01614; A0A087WW87	IGKV2-40; IGKV2D-40	Immunoglobulin kappa variable 2–40
O15031	PLXNB2	Plexin-B2

## Discussion

This study employed a strong research design to investigate the effects of 2-weeks intake of a mixed beet-based supplement (212 mg nitrates per day) on inflammation induced by a 2.25 h cycling bout with 20 cyclists. A multi-omics approach was used, and this included a comprehensive targeted oxylipins panel and the measurement of several hundred proteins using untargeted proteomics. BEET supplementation significantly increased plasma NO_3_^−^ + NO_2_^−^ 1.6-fold after the 2-week supplementation period and 2.6-fold above placebo levels immediately after the cycling bout. The 2.25 h cycling bout caused significant inflammation with a composite variable of 41 oxylipins rising 3.4-fold immediately post-exercise above baseline plasma levels. Plasma oxylipins were still elevated 1.5-fold at the 3 h-post-exercise time point before falling to near baseline levels after 24 h recovery. BEET supplementation did not reduce post-exercise plasma concentrations of pro-inflammatory oxylipins but did have a modest effect in elevating two anti-inflammatory EPA- and DHA-derived oxylipins (18-HEPE and 4-HDoHE). Two-way ANOVA revealed 66 proteins that were increased or decreased with BEET supplementation, and functional enrichment supported significant BEET-related reductions in inflammation-related proteins including proteins related to complement activation, the acute phase response, and immune cell adhesion, migration, and differentiation.

This is the first exercise-based human clinical trial to investigate the influence of BEET supplementation on inflammation using a human systems biology approach. Other similar studies used a few targeted outcomes, had widely disparate research designs, and generally reported null or minor effects of increased plasma NO_3_^−^ + NO_2_^−^ from nitrate supplementation on post-exercise inflammation biomarkers including cytokines (8, 17–19). In one study with 34 runners, beetroot juice versus placebo supplementation for 3 days following a marathon race had no effect on muscle soreness, creatine kinase, interleukin (IL)-6, IL-8, tumor necrosis factor-α (TNFα), blood leukocyte cell counts, or C-reactive protein (CRP) (19). The major limitation was that beetroot juice supplementation was restricted to the 3-day period after running the marathon race when most of the inflammation biomarkers were similar to pre-marathon levels.

Nearly all inflammation regulatory oxylipins generated in response to exercise stress come from PUFA substrates that are released from cell membranes and subsequently oxidized from CYP, LOX, and COX enzyme systems (20). Oxylipin production during recovery from prolonged and intensive cycling is sensitive to nutritional interventions including carbohydrate and blueberry supplementation (20–24). Underlying mechanisms are still being explored but may include the influence of glucose and polyphenol metabolites on CYP, LOX, and COX enzyme activity and the subsequent generation of specific oxylipin subgroups (21–24). Nitric oxide in cell culture and rodent-based studies inhibits P450 and LOX enzymes through multiple pathways, and can inhibit or stimulate COX enzymes depending on the physiological context (35, 36). Nitric oxide and oxylipins are viewed as interdependent signaling systems that help regulate inflammation, immune, and metabolic responses (37). Human clinical trials focused on nitrate supplementation and inflammation regulation from nitric oxide and oxylipins, however, are lacking.

In our study, BEET supplementation had a modest effect in increasing post-exercise EPA-COX derived HEPE-18 and DHA-LOX derived 4-HDoHE but had no significant effects on the generation of pro-inflammatory linoleic acid (LA)-CYP, LA-LOX, or arachidonic (ARA)-CYP oxylipins. These results suggest that pro-inflammatory oxylipin generation after 2.25 h intensive cycling is relatively unaffected by increases in plasma NO_3_^−^ + NO_2_^−^ with 2-weeks BEET intake. Whether or not higher and/or longer duration BEET or nitrate dosing may alter these results remains to be determined. In two previous studies, we showed that 14–18 days supplementation with 1 cup equivalent of blueberries had significant effects on post-exercise plasma oxylipin concentrations (21, 23). In one of these studies, blueberry ingestion was linked to a sustained elevation in DHA- and EPA-derived anti-inflammatory oxylipins including 18-HEPE in response to a 90-min bout of unaccustomed exercise by untrained adults (21). 18-HEPE is a precursor for resolvins that are specialized pro-resolving lipid mediators (SPM) with well-defined roles in inflammation resolution (38). 4-HDoHE is a beneficial 5-LOX oxidation product from DHA. Limited data indicate that this oxylipin in cell culture exerts anti-oxidative and anti-inflammatory effects on brain and blood vessel tissues through nuclear factor erythroid 2–related factor 2 (Nrf2) activation (39, 40).

Previous studies using untargeted proteomics methods indicate that acute prolonged and intensive exercise bouts have a widespread effect on plasma concentrations of proteins involved in immune system and inflammation responses (22, 25–27, 41, 42). The use of untargeted proteomics in studies with a sports nutrition focus is an emerging research design approach (22, 43). In a recent untargeted proteomics-based study, our research group showed that 4-weeks supplementation with the keto-carotenoid astaxanthin (8 mg/day) countered post-exercise decreases in humoral immunity and plasma immunoglobulins in athletes engaging in a vigorous 2.25 h running bout (22). In the current study, untargeted proteomics revealed an extensive effect of BEET versus placebo supplementation on 66 proteins, with lower plasma levels for a cluster of 45 proteins and higher levels for a cluster of 21 proteins. Functional enrichment supported a significant reduction in exercise-induced complement activation and inflammatory and stimulus responses. Key inflammation-related proteins that were lower with BEET supplementation included complements C3, C5, and C9, properdin (a positive regulator of the alternate pathway of complement), ficolin-3 (innate immune activator through the lectin complement pathway), serum amyloids A1 and P (acute phase proteins), alpha-1-microglobulin (an inflammation biomarker with acute phase proteins), clusterin (diverse inflammation roles), fibulin-1 and fermitin family homolog 3 (both involved with cell adhesion, migration, and differentiation), hypoxia up-regulated protein 1 (heat shock protein), coronin-1C (plays an important role in immune cell motility and vesicle trafficking), peroxiredoxin-1 (anti-oxidant that can promote inflammation), peroxisome proliferator-activated receptor-delta (regulates cellular metabolic functions and can promote inflammation), reelin (pro-inflammatory and pro-thrombotic factor), and dipeptidyl peptidase 4 membrane form (regulator of T-cell proliferation and natural killer cell kappa B activation). The liver is the source of most complement and acute phase proteins, and complement activation after prolonged and intensive exercise is an acute phase response to physiological stress (44). Nitric oxide is a pleiotropic and pervasive signaling molecule and influences inflammation in multiple ways including the suppression of immune cell growth, cytokine production, and platelet activation, and regulation of NF-κB activation (45).

Table 5 lists 14 immunoglobulin proteins that were lower with BEET versus placebo supplementation. Functional enrichment through STRING does not include immunoglobulins. Although human data are limited, emerging results from animal-based studies indicate an inverse relationship between nitric oxide and immunoglobulin production (46–48). Elevated nitric oxide may have multiple negative influences on B cell function. Nitric oxide generated from inflammatory monocytes, for example, can decrease B cell survival and hence antibody production (47). Vaccine adjuvants are being developed to counter the negative effect of nitric oxide on antibody responses (48). Our finding that BEET intake was associated with lower immunoglobulin proteins in cyclists during recovery from vigorous exercise was unexpected and needs to be confirmed in future studies and then evaluated for clinical significance.

BEET supplementation was linked to an increase in proteins involved with regulation of insulin like growth factors (IGFs) and symbiotic interactions. The IGF-axis mediates many of the physiological effects of growth hormone and is involved in the regulation of cell growth, proliferation, and survival (49). IGF binding protein 2 (IGFBP2) modifies IGF-1 functions and this protein was elevated with BEET supplementation. Nitric oxide mediates some of the effects of IGF-1 on vasodilation, increased blood flow, and lowered blood pressure (50). Other proteins with symbiotic interactions were elevated with BEET supplementation including neural cell adhesion molecule 1 (NCAM1 or CD56), heat shock cognate 71 kDa protein (HSPA8), and annexin A5. These proteins contribute to many biological processes including cell growth and differentiation, signaling, protein homeostasis, and apoptosis (51–53). Alpha-2-HS-glycoprotein or fetuin-A is a multifunctional protein, has a complex involvement with inflammation depending on the physiological context, and can function as an anti-inflammatory acute phase protein (54). Intercellular adhesion molecule 1 (ICAM-1) is an adhesion receptor and was also elevated with BEET supplementation. ICAM-1 regulates inflammatory responses by controlling leukocyte recruitment from the blood compartment to sites of inflammation and also is involved in epithelial injury-resolution responses (55).

The strength of this study was the combination of a strong research design and multi-omics outcomes to determine if exercise-induced inflammation could be moderated with BEET supplementation. There are several research design features and limitations that should be considered when interpreting study results. This study included non-elite male and female cyclists who were generally middle-aged and cycled for 2 h 15 min at about 75% VO_2max_. Thus, the results of this study may not apply to younger elite endurance athletes who exercise at higher intensities. The research design included a 2-week supplementation period of BEET or placebo (two doses per day) with intake continued the day of the exercise challenge. Thus, the changes in protein biomarkers reported in this study could be related to both acute and chronic BEET intake. This research design element is consistent with the practice of many athletes who consume supplements both during training and on the day of competition. Plant-based supplements undergo a complex metabolic process involving the oral and gut microbiomes, metabolite formation from substrates, and subsequent biochemical effects on various enzyme systems including those related to inflammation. Our blood samples were collected in an overnight fasted state (pre- and post-supplementation). On the exercise challenge day, the blood collection was followed by the normal BEET dose and then the cycling bout. Thus, subjects had not consumed the BEET or placebo supplement since the prior day (midday) and most likely explains the low pre-exercise plasma NO_3_^−^ + NO_2_^−^ levels. The primary purpose of this study was to investigate the effects of BEET supplementation on exercise-induced inflammation. For this reason, participants exercised in an overnight fasted state to avoid the interactive effects of carbohydrate intake on inflammation. The BEET supplement included a moderate amount of nitrate from beets and fermented beets with caffeine, vitamin C, and other food components that may minimize risks from N-nitroso compounds. Beetroot powder also contains other phytochemicals that may have bioactive effects. We cannot rule out the potential synergistic effects of these other ingredients with beetroot on inflammation.

## Conclusion

This randomized crossover study used a 2.25 h cycling bout to induce inflammation. Using double blind, placebo-controlled methods, 2-weeks intake of a BEET-based supplement with a moderate amount of nitrate (212 mg/d) increased plasma levels of NO_3_^−^ + NO_2_^−^. BEET supplementation did not counter exercise-induced increases in pro-inflammatory oxylipins but did increase post-exercise levels of two anti-inflammatory EPA- and DHA-derived oxylipins. The strongest effect of BEET supplementation was related to a significant decrease in a cluster of proteins involved in complement activation and inflammation. These data support that 2-weeks intake of a mixed beet-based supplement with morning and midday doses moderates protein biomarkers of exercise-induced inflammation in athletes. Carbohydrate and blueberry ingestion also mitigate post-exercise inflammation, an effect that requires more investigation for biological and clinical significance, but is generally interpreted as beneficial to the athlete over the long term (21, 23, 24, 29). This study did not focus on testing different formulations or dosing approaches. Future research can establish whether higher beet-nitrate intake with various types of adjuvants over a longer time period can amplify the anti-inflammatory effects, especially on pro-inflammatory oxylipins.

## Data availability statement

The datasets presented in this study can be found in online repositories. The names of the repository/repositories and accession number(s) can be found in the article/Supplementary material.

## Ethics statement

The studies involving humans were approved by Appalachian State University Institutional Review Board, NIH. The studies were conducted in accordance with the local legislation and institutional requirements. The participants provided their written informed consent to participate in this study.

## Author contributions

DN: Conceptualization, Formal analysis, Funding acquisition, Investigation, Methodology, Project administration, Resources, Supervision, Writing – original draft, Writing – review & editing. CS: Conceptualization, Formal analysis, Funding acquisition, Investigation, Methodology, Project administration, Resources, Supervision, Writing – review & editing. JW: Formal analysis, Investigation, Methodology, Writing – review & editing. FM: Data curation, Formal analysis, Methodology, Writing – review & editing. PS: Data curation, Formal analysis, Investigation, Methodology, Writing – review & editing. AO: Data curation, Formal analysis, Investigation, Writing – review & editing. QZ: Conceptualization, Data curation, Formal analysis, Investigation, Methodology, Project administration, Supervision, Writing – review & editing.

## Footnotes

^1^ https://string-db.org/
